# Quantum AI simulator using a hybrid CPU–FPGA approach

**DOI:** 10.1038/s41598-023-34600-2

**Published:** 2023-05-12

**Authors:** Teppei Suzuki, Tsubasa Miyazaki, Toshiki Inaritai, Takahiro Otsuka

**Affiliations:** Research and Development Center, SCSK Corporation, Toyosu Front, 3-2-20 Toyosu, Koto-ku, Tokyo, 135-8110 Japan

**Keywords:** Engineering, Physics

## Abstract

The quantum kernel method has attracted considerable attention in the field of quantum machine learning. However, exploring the applicability of quantum kernels in more realistic settings has been hindered by the number of physical qubits current noisy quantum computers have, thereby limiting the number of features encoded for quantum kernels. Hence, there is a need for an efficient, application-specific simulator for quantum computing by using classical technology. Here we focus on quantum kernels empirically designed for image classification and demonstrate a field programmable gate arrays (FPGA) implementation. We show that the quantum kernel estimation by our heterogeneous CPU–FPGA computing is 470 times faster than that by a conventional CPU implementation. The co-design of our application-specific quantum kernel and its efficient FPGA implementation enabled us to perform one of the largest numerical simulations of a gate-based quantum kernel in terms of features, up to 780-dimensional features. We apply our quantum kernel to classification tasks using the Fashion-MNIST dataset and show that our quantum kernel is comparable to Gaussian kernels with the optimized hyperparameter.

## Introduction

Quantum computing^1^ is an emerging technology that could transform many areas of industries and scientific research, including finance^2^, chemistry^3^, and machine learning (ML)^4,5^. In particular, quantum machine learning (QML)^4–17^ has received considerable attention at a rapid rate, indicating that QML is a plausible candidate for the practical application of near-term quantum devices. While early fault-tolerant quantum computing has been demonstrated recently^18^, noisy intermediate-scale quantum (NISQ) processors are currently available through various hardware platforms with $\sim$ 10–100 physical qubits. However, the number of physical qubits today’s NISQ computers have is generally insufficient to explore practical applications of QML. Therefore, there is a need for application-specific quantum computing simulators to explore and validate the practicality of QML in real-world settings.

The quantum kernel method is a NISQ algorithm in the framework of the hybrid quantum–classical approach^19,20^ and can also be feasible on current NISQ computers with shallow quantum circuits^9,12,13,16,17^. In the quantum kernel method, a quantum feature map can be described explicitly by a quantum circuit and the quantum kernel entry can be estimated by measuring the inner product of the quantum feature map^8,9^. The calculation of quantum kernels when using real devices or general-purpose simulators based on quantum assembly language (QASM) requires a number of measurements to obtain the quantum kernel entries (note that measurements are a key part of the QASM simulator, which handles measurements by collapsing the state of the qubit according to the probabilities predicted by quantum mechanics). Commonly used quantum kernels inspired by instantaneous quantum polynomials (IQP)^9^ can be computationally prohibitive on classical computers as the number of qubits increases; for instance, the number of entangled qubits in the simulation of quantum kernels using state-of-the-art classical platforms is 30^11^. On the other hand, it becomes challenging to reliably estimate such quantum kernels using near-term quantum devices with increasing size in circuits, owing to expensive gate costs, low gate fidelities, and different qubit connectivities. The above points can be a drawback in exploring practical applications of quantum kernels since machine learning models typically improve performance by increasing training data samples or expanding the number of input features. There is still a gap between theoretical developments and practical applications in the quantum kernel method.

To bridge the gap between theory and practice in the quantum kernel method, in this paper, we focus on an application-specific quantum kernel that can be applied to image data with a large number of features. To this end, we demonstrate an implementation of an efficient quantum AI simulator by using a heterogeneous classical computing platform. Our approach is highly customized for our specific tasks at the hardware level and the main objective of our simulator differs from a general-purpose quantum simulator, which is designed to be versatile and to perform a range of quantum algorithms. Until now, there have been considerable efforts to develop quantum computing simulators^21–27^. Among hardware implementations, field programmable gate arrays (FPGA) are one of the desirable platforms, because FPGA has the properties of efficient parallelism, low latency, and customization. FPGAs comprise programmable logic blocks that can be interconnected to perform parallel processing, allowing each logic block to perform a specific task simultaneously. FPGAs can also be customized to perform specific tasks using hardware description languages such as Verilog. Herein, we co-design application-specific quantum kernels and our FPGA architecture, which allows efficient numerical simulations. FPGA has been successfully applied to fault-tolerant quantum algorithms such as Grover’s algorithm^28–30^, quantum Fourier transform^28–31^, and Deutsch’s algorithm^32^. However, an FPGA implementation of quantum kernels has been unexplored and the present study is the first demonstration of a gate-based quantum kernel simulator using an FPGA platform.

The rest of the paper is organized as follows. We provide a brief introduction to support vector machine (SVM) and describe our quantum feature map that is useful for image classification. Then we explain the overview of our quantum AI simulator using a heterogeneous CPU–FPGA computing. From an algorithmic point of view, the quantum kernel method can be divided into the quantum kernel estimation and the rest of the tasks. The simulation of the quantum kernel can be computationally demanding; hence, the workload can be accelerated by FPGA hardware. On the other hand, the rest of the tasks, such as dimensionality reduction and the optimization of machine learning parameters, can be efficiently performed on the CPU using existing classical libraries. The FPGA implementation of the quantum kernel is checked in terms of both numerical precision and hardware acceleration. We apply our quantum kernel simulator to binary and multiclass classification for a range of input features using the Fashion-MNIST dataset. Then we summarize our conclusions.

## Results

### Quantum support vector machine

The quantum kernel method is one of the most important algorithms in QML techniques and many studies have been reported^4,5,8,9,11–17^. In the classical kernel method^33,34^ the inner product of the feature map is represented by kernel functions, which implicitly use the Hilbert space; on the other hand, the quantum kernel explicitly defines a quantum feature map by means of a quantum state $\left|\phi ({\varvec{x}})\rangle \right.$ for $d$-dimensional input vectors ${\varvec{x}}\in {\mathbb{R}}^{d}$. The quantum kernel matrix $K\left({\varvec{x}},{\varvec{x}}^{^{\prime}}\right)$ can be estimated by calculating the inner product of the quantum feature map^8,9^:

1 $$K\left({\varvec{x}},{{\varvec{x}}}^{\boldsymbol{^{\prime}}}\right)={\left|\left\langle \phi ({\varvec{x}})|\phi ({\varvec{x}}^{^{\prime}})\right\rangle \right|}^{2}.$$

For binary classification in the framework of SVM, one can obtain a support vector classifier that estimates the label for a new datum ${\varvec{x}}$:

2 $$y = {\text{sgn}}\left( {\sum\limits_{I} {y_{i} \alpha _{i}^{*} K\left( {\varvec{x}^{{\left( i \right)}} ,\varvec{x}} \right) + b^{*} } } \right),$$

where ${y}_{i}\in \left\{+1, -1\right\}$ and parameters ${\{\alpha }_{i}^{*}\}$ and ${b}^{*}$ are the optimal parameters obtained in the training phase^34^. In the hybrid quantum–classical algorithm, the training phase can be performed on classical computers, whereas the quantum kernel entries can be computed by NISQ computers or quantum computing simulators; such methodology is called the quantum SVM (QSVM). The NISQ computation of the quantum kernel requires many quantum measurements to obtain a quantum kernel entry with statistically reliable accuracy. For example, a value for each computational-basis measurement is zero or one. For each quantum kernel entry, $\mathcal{O}\left({N}^{2}\right)$ shots are required with respect to the number of data samples $N$, resulting in the computational complexity of $\mathcal{O}\left({N}^{4}/{\varepsilon }^{2}\right)$ operations with the maximum error $\varepsilon$, in order to obtain all the quantum kernel entries^9^. Such computational complexity prohibits us from developing and validating quantum kernels as the number of data samples grows. Also, the number of entangling qubits with different connectivities in the previously proposed quantum kernels is increased with qubit count^9^, which requires additional computational resources.

To address these issues, here we introduce a shallow, fixed-depth quantum circuit that can be applied to a quantum kernel for a larger number of input features. In the previously proposed quantum kernels based on IQP circuits^9^, the number of dimensional features is typically set to the number of entangled qubits^9,11,14,15^. IQP circuits are a subclass of quantum circuits that cannot be classically efficiently simulated unless the polynomial-time hierarchy collapses to the third level^35^. Here, an IQP circuit is a circuit where a Hadamard gate is applied to each qubit at the beginning and end of the computation, but the rest of the gates are diagonal. In the context of the quantum kernel method, researchers have typically used a more specific type of IQP, called the *ZZ* feature map^9^. In the *ZZ* feature map, the connectivity of qubits is achieved in a pair-wise manner, resulting in $n(n-1)/2$ interactions, where $n$ is the number of qubits. This leads to a rapid expansion of expressibility and results in a deterioration of generalization performance as qubit count increases^11,14,15^. Our approach aims to simplify the quantum feature map, limit the extent to which qubits are entangled, and control the capacity of our QML model, while increasing the number of input features. This framework can handle several hundreds of input features in QSVM. For the $mn$-dimensional input vector ${\varvec{x}}={\left[{{\varvec{s}}}_{1},{{\varvec{s}}}_{2},\cdots ,{{\varvec{s}}}_{m}\right]}^{\mathrm{T}}\in {\mathbb{R}}^{mn}$, where ${{\varvec{s}}}_{b}$ is the $n$-dimensional vector ${{\varvec{s}}}_{b}={\left[{s}_{b, 1},{s}_{b,2},\cdots ,{s}_{b,n}\right]}^{\mathrm{T}}$, we consider a block product state (BPS) wavefunction^36^:

3 $$\left| {{\Psi }^{{{\text{BPS}}}} \left( {\varvec{x}} \right)\rangle} \right. = \left| {\psi_{1} \left( {{\varvec{s}}_{1} } \right)\rangle} \right. \otimes \left| {\psi_{2} \left( {{\varvec{s}}_{2} } \right)\rangle} \right. \otimes \cdots \otimes \left| {\psi_{m} \left( {{\varvec{s}}_{m} } \right)\rangle} \right.,$$

where

4 $$\left|{\psi }_{b}\left({{\varvec{s}}}_{b}\right)\rangle \right.=\left({\otimes }_{q=1}^{n}{R}_{z}\left({s}_{b,q}\right)\right){U}_{{2}^{n}}^{\mathrm{ent}}\left({\otimes }_{q=1}^{n}\left({R}_{y}\left({s}_{b,q}\right){R}_{z}({s}_{b,q})H\right)\right)\left|{0}^{\otimes n}\rangle \right.,$$

and

5 $${U}_{{2}^{n}}^{\mathrm{ent}}:=\prod_{q=1}^{n-1}{\mathbf{C}\mathbf{N}\mathbf{O}\mathbf{T}}_{q,q+1}.$$

In the BPS wavefunction, a modest number of qubits can be entangled within each block (in our numerical simulations, $n$ was varied from 2, 3, and 6); and for the wavefunction $\left|{\psi }_{b}\left({{\varvec{s}}}_{b}\right)\rangle \right.$, each component ${s}_{b,q}$ is encoded three times as the input angle for the ration operator gates (i.e., ${s}_{b,q}$ is encoded in the ${R}_{z}$ gate, in the ${R}_{y}$ gate, and again in the ${R}_{z}$ gate in Eq. (4)). Such kind of redundant encoding leads to the better performance of QML models based on angle encoding^37^. The state $\left|{\psi }_{b}\left({{\varvec{s}}}_{b}\right)\rangle \right.$ is related to matrix product states, a class of tensor networks that have been used for the study of ground states of quantum systems and recently for machine learning. The connectivity of qubits in Eq. (4) is limited to their nearest neighbors, resulting in $(n-1)$ interactions. The idea of BPS has been originally used for ML models based on tensor networks^36^; yet, to our knowledge, this kind of BPS-based quantum feature map has not been applied to quantum kernels. In this work, we will show that such a feature map can be used for QSVM. The kernel associated with the quantum feature map defined by Eq. (3) can be given by

6 $$K\left({{\varvec{x}}}^{(i)},{{\varvec{x}}}^{(j)}\right)={\left|\left\langle {\Psi }^{\mathrm{BPS}}\left({{\varvec{x}}}^{(i)}\right)|{\Psi }^{\mathrm{BPS}}\left({{\varvec{x}}}^{(j)}\right)\right\rangle \right|}^{2}=\prod_{b=1}^{m}{\left|\left\langle {\psi }_{b}\left({{\varvec{s}}}_{b}^{(i)}\right)|{\psi }_{b}\left({{\varvec{s}}}_{b}^{(j)}\right)\right\rangle \right|}^{2}.$$

The number of blocks $m$ can be varied in order to allow a larger number of input features depending on different datasets. Another interesting aspect is that the quantum kernel is not translation invariant, which means that the quantum kernel does not depend solely on the distance of input vectors, in contrast with Gaussian kernels. A computational benefit of our approach is that the calculation of the quantum kernel can be divided into $m$ computational tasks, allowing an efficient computation on classical computers. In particular, ${\left|\langle {\psi }_{b}^{i}|{\psi }_{b}^{j}\rangle \right|}^{2}$ in Eq. (6) can be computed separately; hence, each task can now be efficiently simulated through FPGA acceleration and the multiplication can then be performed on CPU.

### Quantum AI simulator using a hybrid CPU–FPGA approach

**Figure 1:**
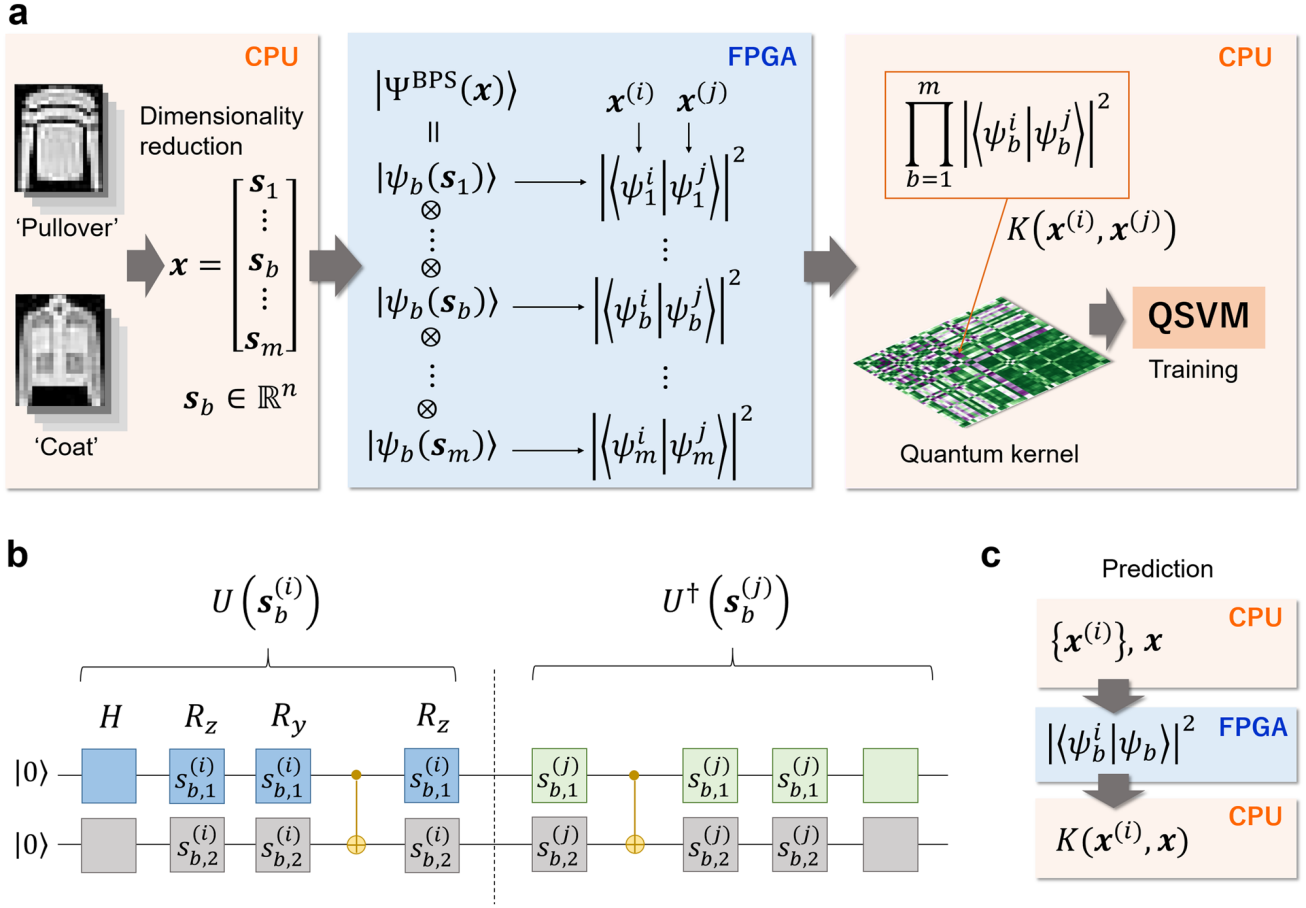
Schematic representation of our quantum AI simulator using a hybrid CPU–FPGA approach. (**a**) PCA is used to reduce the dimension of the original data from Fashion-MNIST; a range of features from $d=4$ to $d=780$ can be used for machine learning. Then PCA-reduced features are sent from the CPU to the FPGA. We calculate the square of the norm of the inner products for each block wavefunction $\left|{\psi }_{b}\right.\rangle$ of the quantum feature map. This process is repeated for all the pairs of the data points (i.e., ${N}^{2}/2$ times). The data are then sent back to the CPU. A kernel matrix value can be obtained by multiplying $m$ blocks. After all the quantum kernel entries are computed, the SVM algorithm is performed on the CPU. (**b**) 2-qubit example of a quantum circuit that performs the estimation of the quantum kernel element. For the entangling gate, the CNOT gate is used. The quantum circuit is simulated on FPGA using the procedure described in the text (see Methods). (**c**) Test process: the decision function can be computed using the hybrid CPU–FPGA scheme. Details of computational resources in the cloud system (FPGA and CPU) are given in Methods.

By co-designing FPGA architecture and a quantum kernel given by a shallow quantum circuit, we implemented a fast and efficient quantum AI simulator using a heterogeneous computing approach (Fig. 1a). Details of computational resources in the cloud system (FPGA and CPU) are given in Methods. To begin with, using the principal component analysis (PCA) method^38^ we conducted the dimensionality reduction of the $28\times 28$ image data from the Fashion-MNIST dataset^39^; then the number of input features can be varied from $d=4$ to $d=780$. After obtaining PCA-reduced input vectors ${{\varvec{x}}}^{(i)}\in {\mathbb{R}}^{d}$, the input data are sent from CPU to the internal memory of an FPGA hardware via PCI express. Then, for each block wavefunction $\left|{\psi }_{b}\left({{\varvec{s}}}_{b}\right)\right.\rangle (b=1,\cdots ,m)$ of the quantum feature map, we calculate the square of the norm of the inner products ${\left|\langle {\psi }_{b}^{i}|{\psi }_{b}^{j}\rangle \right|}^{2}$ (which is depicted in Fig. 1b) on our FPGA architecture in the following procedure: First, the sine and cosine of the input angles for quantum gates are computed using the COordinate Rotational DIgital Computer (CORDIC) algorithm^40^. Second, the square of the norm of the inner product can be calculated using the unitary matrices in Eq. (4), together with an efficient implementation of $n$-qubit entanglement. (The procedure is described in great detail in Methods and Supplementary Notes 1 and 2.) This process can be repeated for all the pairs of data samples, namely, for ${N}^{2}/2$ cycles. The processed, real-valued data are sent back to the CPU. The kernel matrix element will thus be calculated by the multiplication of $m$ blocks. After all the kernel entries are obtained, the training phase of the SVM can be performed on the CPU platform. In the test process (Fig. 1c), prediction can be done using the same FPGA acceleration with $\mathcal{O}(ND)$ operations, where $D$ is the number of test data.

### FPGA implementation: numerical precision and acceleration

**Figure 2:**
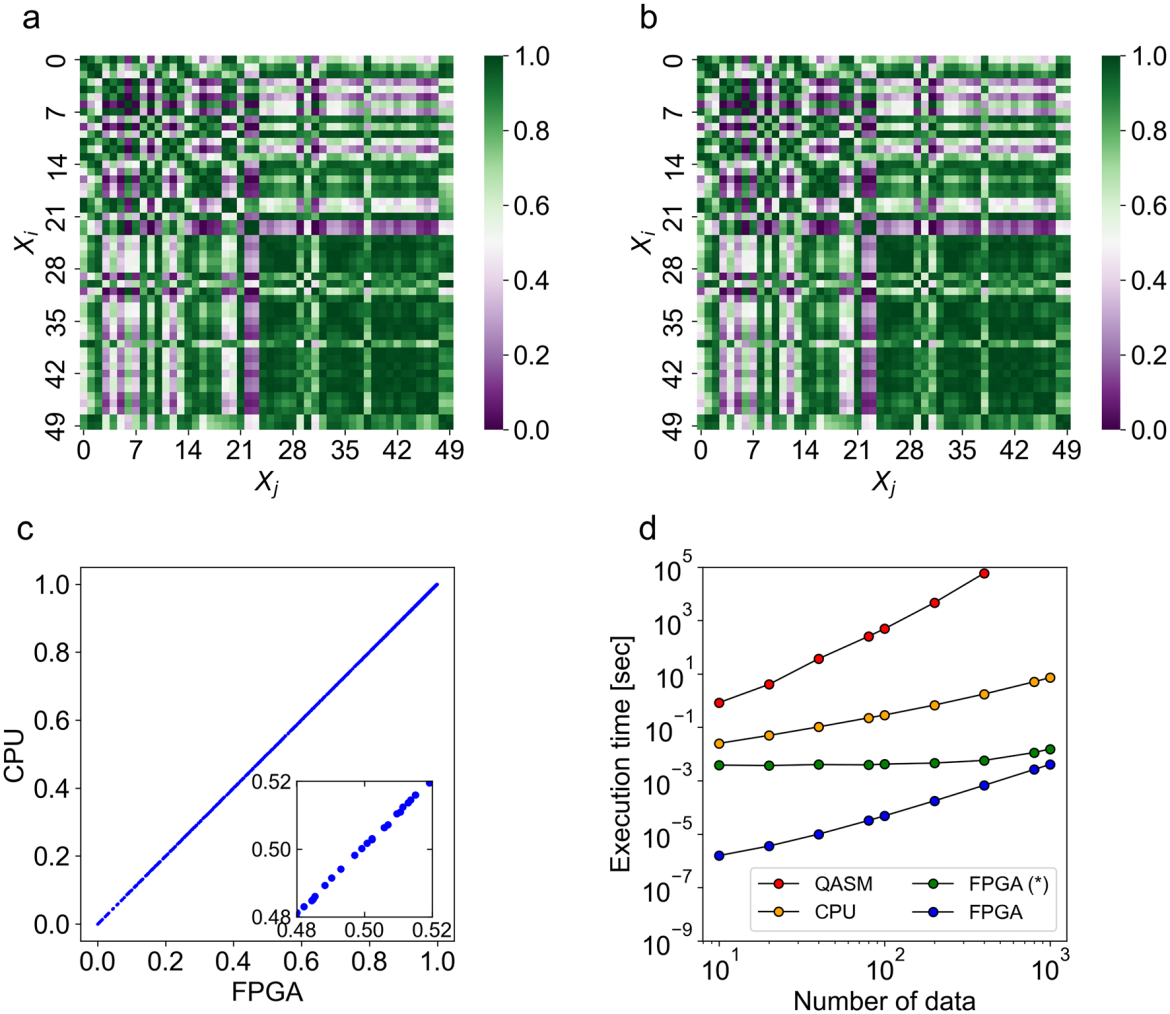
FPGA implementation of the quantum kernel and its execution time. The numerical simulations were performed on a 6-qubit quantum circuit that estimates the quantum kernel element. (**a**) Quantum kernel matrix obtained by an FPGA platform (16-bit fixed-point arithmetic). (**b**) Quantum kernel matrix obtained by a CPU platform (64-bit floating-point arithmetic). (**c**) Parity plot for the quantum kernel values obtained by CPU and FPGA platforms. Inset shows small differences between the two: the error between the two hardware platforms was ± $\sim$ 0.095%. (**d**) Execution time with respect to the number of data $N$ for different platforms: FPGA, blue; FPGA (including CPU–FPGA communication overhead; denoted by the asterisk), green; CPU, orange; QASM quantum simulator (Qiskit Aer), red. Note that the FPGA execution including communication overhead was 472 times faster than that for the CPU counterpart at $N=1000$.

Herein we validate our FPGA implementation in terms of numerical precision and acceleration. We begin by comparing the quantum kernel values obtained by the FPGA platform and those obtained by the CPU platform (Fig. 2a–c). The norms of inner products ${\left|\langle {\psi }_{b}^{i}|{\psi }_{b}^{j}\rangle \right|}^{2}$ have values between 0 and 1. Such property along with a shallow circuit depth is amenable to the use of 16-bit fixed-point arithmetic in our FPGA architecture, which in turn makes the calculation faster with efficient hardware utilization. We also employed 64-bit floating-point arithmetic in the CPU platform to validate our FPGA implementation. The parity plot suggests the success of our FPGA implementation of the quantum kernel (Fig. 2c). The numerical deviation between the two hardware platforms was ±$\sim$ 0.095%, indicating that there was a negligible loss of numerical accuracy.

Next, we compare the execution time for computing a kernel matrix (in the case of 6 entangled qubits) using the FPGA platform with the one obtained by our CPU implementation, as well as the one obtained by Qiskit Aer^21^, a QASM quantum computing simulator (Fig. 2d). Measurement is a vital aspect of the simulation process in the QASM simulator, which handles measurements by collapsing the state of the qubit according to the probabilities determined by the state of the qubit. Therefore, in the QASM simulator, a number of shots are required to obtain the expectation value. In our CPU implementation, the kernel matrix entry is obtained directly by calculating the inner product of the state vectors. In particular, we used NumPy^41^, which is a popular library for scientific computing and data analysis (note that the core of NumPy is implemented in C Language). For our particular tasks (in the case of 6 entangled qubits), the execution time by our CPU implementation is likely to be somewhat faster than that by the state-vector simulator; this is because the state-vector simulator tracks the quantum state of the system as it evolves through the circuit, resulting in a slowing down of the execution time. Thus, the plot for the state-vector simulation would be the upper side of the plot denoted by orange in Fig. 2d.

In our FPGA architecture, once the data are sent to the FPGA architecture, we used only the internal memory of the FPGA hardware without accessing the external (off-chip) memory, which circumvents the associated communication overhead (for more details of our FPGA architecture, see Supplementary Note 2). In addition, two more factors are responsible for the FPGA acceleration. First, an FPGA allows each programable logic block to perform a specific task simultaneously in an efficient manner. Second, an FPGA can be customized to perform specific tasks using the hardware description language, resulting in faster performance in comparison with CPUs.

In our FPGA implementation, all the kernel entries were computed in 4.1 ms at $N=1000$; and the execution time including CPU–FPGA communication overhead was 15.4 ms at $N=1000$. In other words, our FPGA implementation achieved a 1784 × improvement in comparison with the CPU counterpart. Also, the execution time including the communication overhead was 472 times faster (Fig. 2d); moreover, in comparison with the execution by a QASM simulator (assuming that the computation cost grows as $\mathcal{O}\left({N}^{4}/{\varepsilon }^{2}\right)$ operations), a 10 million times speedup was accomplished at $N=400$ (Fig. 2d). The results show that our FPGA implementation is highly efficient in terms of the number of data samples, with a modest number of entangling qubits (up to 6 qubits) being used in our quantum feature map. Owing to the limitation of the internal memory and digital single processors within an FPGA, however, our implementation technique will be prohibitive for $n$ large than 8. Nonetheless, for our machine-learning tasks, this can be overcome by dividing input features into a number of blocks, and each block’s quantum kernel can be efficiently computed in FPGA. Thus, the FPGA-based simulator accelerates the numerical simulations of QSVM using our quantum kernel and allows us to validate its applicability to much larger features in quantum kernel methods.

### Binary classification on Fashion-MNIST dataset

**Figure 3:**
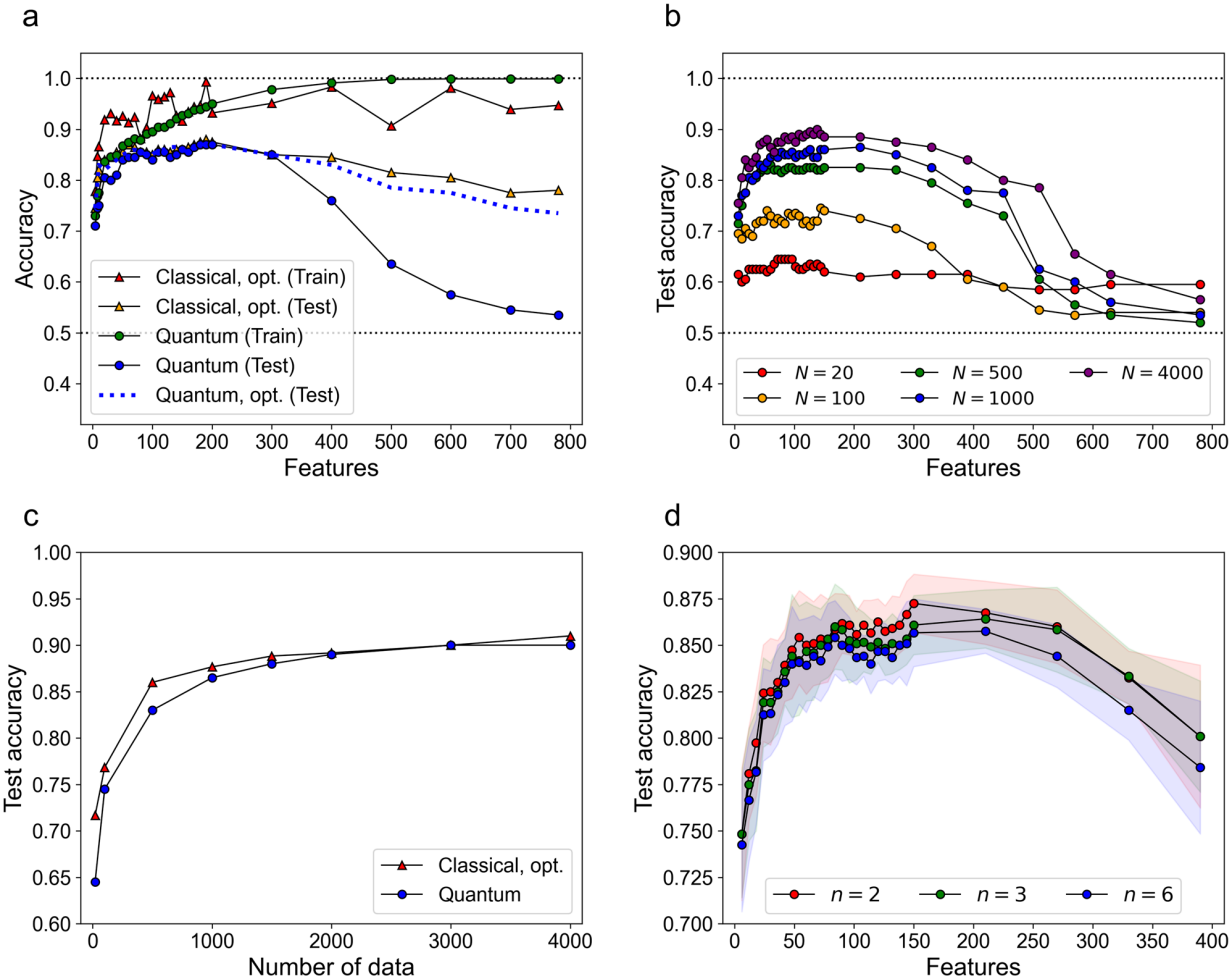
Train and test accuracies of the quantum kernel on the Fashion-MNIST dataset. (**a**) Training and test accuracies with a range of features from $d=4$ to $d=780$ using 1000 data samples. The coat versus shirt (4 vs. 6) classification task was used. The performance of the Gaussian kernel with the optimized bandwidth for each dimension (train: red triangle; test: yellow triangle) is compared with that of the quantum kernel (train: green circle; test: blue circle). Note that the performance of our quantum kernel without introducing any hyperparameter is comparable to that of the classical kernel with the optimized bandwidth for the dimension $d$ smaller than $\sim$ 300. Introducing the scaling parameter improved the performance of our quantum kernel for the dimension $d$ larger than $\sim$ 300, which is indicated by the blue dotted line. (**b**) Test accuracies obtained by the quantum kernel for a range of features with varying the number of data samples ($N=20, 100, 500, 1000, 4000$). The results were averaged over the three tasks: pullover versus shirt (2 vs. 6), pullover versus coat (2 vs. 4), and coat versus shirt (4 vs. 6) classification tasks. (**c**) Best test accuracies with respect to the number of data samples $N$, up to $N=4000$ for the best classical (red triangle) and the quantum (blue circle) kernels. Each plot represents the best test accuracy for a given $N$. The results were averaged over the same three tasks as in (**b**). (**d**) The effect of quantum entanglement within a block (the block size $n=2, 3, 6$). The coat versus shirt (4 vs. 6) classification task was used. The shaded regions indicate the standard deviation over 6 independent runs.

 Having shown the accuracy and efficiency of our FPGA-based quantum kernel estimation, we now turn to the performance of our quantum kernel. To begin with, we trained classical and quantum SVMs on Fashion-MNIST and obtained 45 binary classifiers. Among 45 pairs of binary classification tasks from 10 categories of Fashion-MNIST^39^ (0, t-shirt/top; 1, trouser; 2, pullover; 3, dress; 4, coat; 5, sandals; 6, shirt; 7, sneaker; 8, bag; 9, ankle boots), about half the pairs of classification tasks were relatively easy to distinguish. On the other hand, more challenging tasks such as pullover versus shirt (2 vs. 6), pullover versus coat (2 vs. 4), and coat versus shirt (4 vs. 6) classification tasks were somewhat difficult to distinguish (e.g., the images of pullovers are more similar to those of coats than to those of trousers). Hence, we focused on the three binary classification tasks and investigate the performance in detail (Fig. 3).

The performance of our quantum kernel without introducing any hyperparameter was comparable to that of the Gaussian kernel $\mathrm{exp}\left(-\gamma {\Vert {{\varvec{x}}}^{(i)}-{{\varvec{x}}}^{(j)}\Vert }^{2}\right)$ with the optimized bandwidth $\gamma$, for dimensions smaller than $\sim$ 300 (Fig. 3a). Here, a key hyperparameter in the Gaussian kernel is the kernel bandwidth $\gamma$, which is known to affect the performance of kernel-based methods such as SVMs and is routinely optimized when SVMs are used in practice. The hyperparameter $\gamma$ controls the smoothness of the decision boundary in the SVM. Analogously, we introduced a scaling hyperparameter $\uplambda$ (i.e., ${{\varvec{x}}}^{(i)}\leftarrow\uplambda {{\varvec{x}}}^{(i)}$ in the quantum circuit) to improve the performance of QSVM. The role of $\uplambda$ appears to be similar to the classical counterpart. The hyperparameter $\uplambda$ can calibrate the angles of the rotation gates and directly affect the quantum feature map in the Hilbert space. From a physical point of view, changing the hyperparameter $\uplambda$ in the quantum kernel is related to changing the total evolution time in the Hamiltonian evolution^15^. The best test accuracy for the quantum kernel was 0.87 at $d=180, 190, 200$; whereas that for the classical kernel with the optimal bandwidth was 0.88 at $d=190$. We found that introducing the scaling parameter $\uplambda$ improved the performance of our quantum kernel for larger dimensions ($d>\sim 300$), maintaining its comparable performance to the classical kernel, which is indicated by the blue dotted line in Fig. 3a (for the grid search over the hyperparameters of the classical and quantum kernels, see Supplementary Note 3).

The test accuracy obtained by our quantum kernel was improved by increasing the number of data samples $N$ (Fig. 3b). In particular, as the number $N$ was increased, the test accuracies for higher dimensional vectors tended to improve gradually (Fig. 3b). But the relatively sharp drop for dimensions higher than $\sim$ 300 was difficult to overcome just by increasing $N$; nonetheless, the dimension $d$ that gave the best test accuracy was typically in the range between 100 and 200 for this particular application. We note that the drop in the test accuracy for higher dimensions can be overcome by optimizing the aforementioned scaling parameter (which will be discussed in multiclass classification).

The performance of our hyperparameter-free quantum kernel was competitive with the Gaussian kernel with the optimized bandwidth at $N>1500$ (Fig. 3c), which might be beneficial for practical applications. The best test accuracies at $N=2000$ and $N=3000$ were 0.89 and 0.90, respectively, for both of the two kernels. For smaller numbers of data samples ($N<1000$), the performance of our quantum kernel was slightly lower than the best classical counterpart. To understand the role of quantum entanglement, we investigated the effects of enlarging the number of entangled qubits. Increasing the number of entangled qubits (from 2 to 6 qubits per block) did not significantly change the performance for PCA-reduced input vectors (Fig. 3d); this kind of insensitiveness to quantum entanglement has been previously reported in an ML model based on tensor networks using BPS^36^. Our results probably indicate that the capacity of our quantum feature map is already sufficiently high even in the case of $n=2$. However, this may not necessarily mean that quantum entanglement is unimportant; the CNOT entangling gate can make the quantum feature map more complex in comparison with no quantum entanglement. Overall, the behavior of our quantum kernel is quite different from the previously used quantum kernels^9,11–15^. The results suggest that our quantum kernel is comparable to the best classical kernel with good generalization performance for a range of features.

### Multiclass classification on Fashion-MNIST dataset

**Figure 4:**
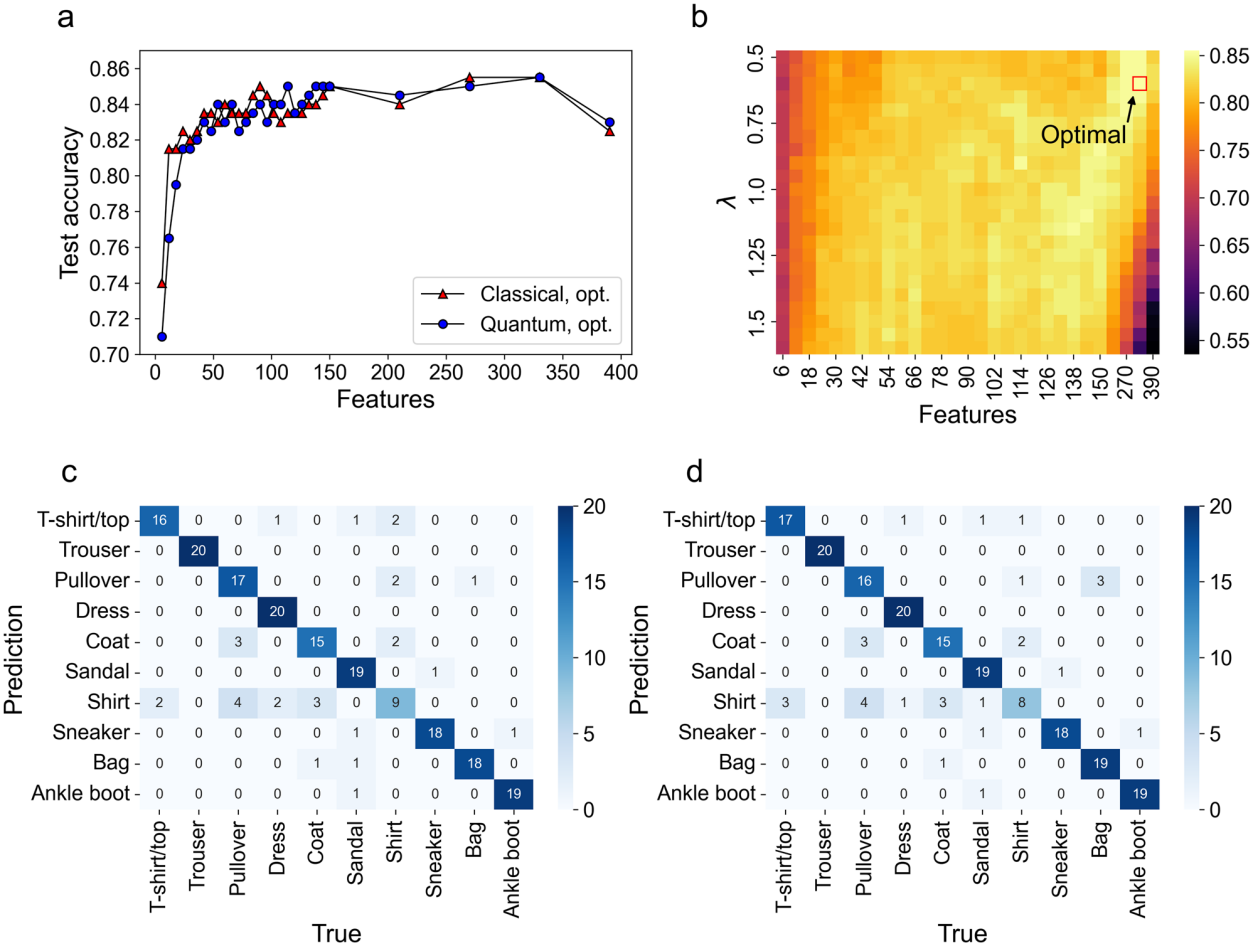
Multiclass classification on Fashion MNIST dataset. (**a**) Test accuracies for a range of PCA-reduced features from $d=4$ to $d=390$ (the block size $n=2$) for the best classical (red triangle) and the quantum (blue circle) kernels. The number of data samples was 1000. For the classical kernel, we used Gaussian kernels with the optimized bandwidth for each dimension. For the quantum counterpart, the optimal scaling parameter $\uplambda$ (${{\varvec{x}}}^{(i)}\leftarrow\uplambda {{\varvec{x}}}^{(i)}$) was used for each dimension. The quantum kernel with the optimal scaling parameter is competitive with the classical counterpart. (**b**) Grid search over the scaling parameter $\uplambda$ for a range of features. The scaling parameter that gave the optimal test accuracy is indicated by the red open square: ${\uplambda }^{*}=0.6$ at $d=330$. (**c**) Confusion matrix obtained from the best classical kernel (dimension $d=330$). The performance metrics were as follows: accuracy, 0.855; precision, 0.853; recall, 0.855; F-measure, 0.851. (**d**) Confusion matrix obtained by the quantum kernel with the optimized scaling parameter (dimension $d=330$). The performance metrics were as follows: accuracy, 0.855; precision, 0.850, recall, 0.855; F-measure, 0.848.

We also show the numerical results for 10-class classification on Fashion-MNIST. We trained our multiclass QSVMs using a one-versus-rest strategy. As was found in binary classification tasks, our quantum kernel was comparable to the best classical kernel (Fig. 4a). For multiclass classification using the quantum kernel, we found that it was important to introduce the scaling parameter. Hence, we performed a grid search for the scaling parameter $\uplambda$ for a range of features ($4<d<340$) (Fig. 4b). The optimal value for $\uplambda$ was 0.6 at $d=330$. On the other hand, the optimal value for $\upgamma$ of the Gaussian kernel was 2.5 at $d=330$. The confusion matrices for QSVM and SVM were similar to each other (Fig. 4c,d). The performance metrics for the quantum (classical) kernel were the following: accuracy, 0.855 (0.855); precision, 0.850 (0.853); recall, 0.855 (0.855); F-measure, 0.848 (0.851). We note that, among 45 pairs generated by 10 categories of Fashion-MNIST, about half the pairs of classification tasks were relatively easy to distinguish; hence, the difference in the test accuracy between the classical and the quantum kernels tended to be decreased. The results suggest that our quantum kernel performed competitively with the best classical kernel in the multiclass classification task.

## Discussion

In this study, we have implemented an application-specific quantum AI simulator using a heterogeneous CPU–FPGA computing, which was achieved by co-designing the FPGA architecture and our quantum kernel. To this end, we have introduced a BPS structure as a quantum feature map for QSVM, where a small number of qubits are entangled in each block. This is the first demonstration of the FPGA implementation of a gated-based quantum kernel. The co-design of the quantum kernel and its efficient FPGA implementation have enabled us to perform one of the largest numerical simulations of QSVM in terms of input features, up to 780-dimensional data. In the literature, one of the largest simulations of quantum kernels in terms of qubit count was performed by Huang et al.^11^. The number of qubits in their study is 30. For our particular study, increasing the number of entangled qubits is not a practical direction. Instead, our strategy is to divide input features into a number of blocks, and each block’s quantum kernel can be efficiently computed in FPGA. By doing this, hundreds of features can be handled. Our approach is highly customized for our specific tasks at the hardware level; the focus of our simulator differs from that of a general-purpose quantum simulator, which is designed to be flexible and to perform various quantum algorithms. An application of our quantum kernel to dimensional features larger than $\sim$ 1000 would be more challenging because off-diagonal kernel values could become much smaller. This limitation is related to our formalism of the quantum kernel, owing to the multiplication of many values that are less than one in Eq. (6). Nevertheless, the FPGA-based quantum kernel simulator has significantly accelerated our numerical simulations and allowed us to validate the applicability to QSVM with hundreds of input features. The quantum circuit presented in this work might have implications for co-designing quantum software and hardware and for developing application-specific quantum computers^42,43^.

We have demonstrated that the FPGA-based quantum kernel simulator was 470 times faster than that obtained by the CPU implementation, without loss of accuracy. The numerical simulations show that our FPGA implementation is highly efficient in terms of the number of data samples (up to 4000), with a modest number of entangling qubits being used in the quantum feature map. We have applied our quantum kernel to image classification using Fashion-MNIST for a wide range of PCA-reduced features. The results suggest that our quantum kernel is comparable to the best classical kernel, with similar generalization performance for binary and multiclass classification tasks. In binary classification, our hyperparameter-free quantum kernel was comparable to the Gaussian kernels; whereas, in multiclass classification, the scaling parameter played a significant role in improving the performance of our quantum kernel, in line with recent studies^15,44^.

Whether quantum kernels could perform better than classical kernels or have a practical advantage in real-world settings is still an open question. Our quantum kernel may be helpful for understanding the applicability of quantum kernels as well as their limitations. While our quantum kernel was applied to classification, the quantum kernel could be used for other kernel-based ML tasks, such as regression, spectral clustering, Gaussian process^17^, and causal discovery^45^. With hundreds of input features being handled in our quantum kernel, other possible applications might include financial data, cheminformatics, and medical data. There is room for improvement in our quantum feature map. For instance, a recent approach based on the automatic design of quantum feature maps^46^ may possibly improve our quantum feature map or reduce the number of quantum gates required. Nonetheless, our results might have implications for developing quantum-inspired algorithms and designing practical quantum kernels in the NISQ era.

## Methods

### FPGA implementation of the quantum kernel

 We describe an approach for efficient simulation of our quantum kernel, which is particularly designed for our FPGA architecture. The quantum kernel is given by the inner product of the quantum feature map, which in principle requires $\mathcal{O}\left({2}^{3n}\right)$ operations, owing to the multiplication of ${2}^{n}\times {2}^{n}$ matrices to generate the quantum feature map. Such computational complexity becomes prohibitive for efficient FPGA implementation of quantum kernels, because FPGA architecture is memory-bound and the number of complex multipliers is limited. For that reason, efficient resource utilization of FPGA was crucial for calculating our quantum kernel. In this work, we employed a shallow quantum circuit so that we were able to calculate the quantum kernel with $\mathcal{O}({2}^{n})$ operations, as we will see below. This enabled efficient parallelization and the use of internal memory in FPGA.

We consider the following quantum state:

7 $$\left| \psi \rangle \right. = \left( {V_{1} \otimes V_{2} \otimes \cdots \otimes V_{n} } \right)U_{{2^{n} }}^{\rm ent} \left( {U_{1} \otimes U_{2} \otimes \cdots \otimes U_{n} } \right)\left| {0^{ \otimes n} \rangle.} \right.$$

where ${U}_{1}, {U}_{2},\cdots ,{U}_{n}$ and ${V}_{1},{V}_{2},\cdots ,{V}_{n}$ are single-qubit gates and ${U}_{{2}^{n}}^{\mathrm{ent}}:={\prod }_{q=1}^{n-1}{\mathbf{C}\mathbf{N}\mathbf{O}\mathbf{T}}_{q,q+1}$ represents $n$-qubit entanglement operation. For the sake of our discussion, it is convenient to rewrite $\left|\psi \rangle \right.$ as ${\varvec{f}}=V{U}_{{2}^{n}}^{\mathrm{ent}}U{{\varvec{f}}}_{0}$ with ${{\varvec{f}}}_{0}$ being a vector ${\left[\mathrm{1,0},\cdots ,0\right]}^{\mathrm{T}}$, where $U: = U_{1} \otimes \cdots \otimes U_{n}$ and $V: = V_{1} \otimes \cdots \otimes V_{n}$. First, we note that, in the calculation of $U{{\varvec{f}}}_{0}$, only the first column of $U$ is needed; hence, $U{{\varvec{f}}}_{0}$ can be obtained without the need for fully conducting tensor operations. By denoting the first column vector of each $2\times 2$ unitary matrix ${U}_{q}$ as ${\left[{\chi }_{1}^{(q)},{\chi }_{2}^{(q)}\right]}^{\mathrm{T}}$ and the first column vector of $U$ as ${\varvec{u}}={\left[{u}_{1},{u}_{2},\cdots ,{u}_{{2}^{n}}\right]}^{\mathrm{T}}\in {\mathbb{C}}^{{2}^{n}}$, then we have

8 $$U{{\varvec{f}}}_{0}={\varvec{u}}=\left[\begin{array}{c}\begin{array}{c}\begin{array}{c}\begin{array}{c}\begin{array}{c}\begin{array}{c}\begin{array}{c}{\chi }_{1}^{(1)}\cdot \cdots \cdot {\chi }_{1}^{(n-2)}\cdot {\chi }_{1}^{(n-1)}\cdot {\chi }_{1}^{(n)}\\ {\chi }_{1}^{(1)}\cdot \cdots \cdot {\chi }_{1}^{(n-2)}\cdot {\chi }_{1}^{(n-1)}\cdot {\chi }_{2}^{(n)}\\ {\chi }_{1}^{(1)}\cdot \cdots \cdot {\chi }_{1}^{(n-2)}\cdot {\chi }_{2}^{(n-1)}\cdot {\chi }_{1}^{(n)}\end{array}\\ {\chi }_{1}^{(1)}\cdot \cdots \cdot {\chi }_{1}^{(n-2)}\cdot {\chi }_{2}^{(n-1)}\cdot {\chi }_{2}^{(n)}\end{array}\\ \vdots \end{array}\\ {\chi }_{2}^{(1)}\cdot \cdots \cdot {\chi }_{2}^{(n-2)}\cdot {\chi }_{1}^{(n-1)}\cdot {\chi }_{1}^{(n)}\end{array}\\ {\chi }_{2}^{(1)}\cdot \cdots \cdot {\chi }_{2}^{(n-2)}\cdot {\chi }_{1}^{(n-1)}\cdot {\chi }_{2}^{(n)}\end{array}\\ {\chi }_{2}^{(1)}\cdot \cdots \cdot {\chi }_{2}^{(n-2)}\cdot {\chi }_{2}^{(n-1)}\cdot {\chi }_{1}^{(n)}\end{array}\\ {\chi }_{2}^{(1)}\cdot \cdots \cdot {\chi }_{2}^{(n-2)}\cdot {\chi }_{2}^{(n-1)}\cdot {\chi }_{2}^{(n)}\end{array}\right].$$

This calculation can be performed by $4\cdot ({2}^{n-1}-1)$ operations using complex multipliers in FPGA (more details are given in Supplementary Fig. 5). The feature map can thus be rewritten as ${\varvec{f}}=V{U}_{{2}^{n}}^{\mathrm{ent}}{\varvec{u}}$. Next, we note that $V$ is a diagonal matrix in our quantum circuit and that ${U}_{{2}^{n}}^{\mathrm{ent}}$ is a sparse matrix, in which each row vector contains only one non-zero entry. By denoting the diagonal elements $\left\{{V}_{kk}\right\}$ as ${\varvec{v}}={\left[{v}_{1},{v}_{2},\cdots ,{v}_{{2}^{n}}\right]}^{\mathrm{T}}\in {\mathbb{C}}^{{2}^{n}}$, we can calculate ${\varvec{f}}$ as

9 $${f}_{k}={v}_{k}{u}_{{\upxi }_{k}}.$$

Here ${\upxi }_{k}$ is the index of the non-zero element in the $i$ th row of ${U}_{{2}^{n}}^{\mathrm{ent}}$ (e.g., for $n=2$, then ${\upxi }_{1}=1$, ${\upxi }_{2}=2$, ${\upxi }_{3}=4$, and ${\upxi }_{4}=3$). In general, ${U}_{{2}^{n}}^{\mathrm{ent}}$ can be calculated recursively by

10 $${U}_{{2}^{n+1}}^{\mathrm{ent}}=\left[\begin{array}{cc}{U}_{{2}^{n}}^{\mathrm{ent}}& {O}_{{2}^{n}}\\ {O}_{{2}^{n}}& {Y}_{{2}^{n}}\end{array}\right];\begin{array}{cc}{Y}_{{2}^{n+1}}=\left[\begin{array}{cc}{O}_{{2}^{n}}& {U}_{{2}^{n}}^{\mathrm{ent}}\\ {Y}_{{2}^{n}}& {O}_{{2}^{n}}\end{array}\right]& (n\ge 1)\end{array}.$$

where ${U}_{2}^{\mathrm{ent}}$ and ${Y}_{2}$ denote the $2\times 2$ identity matrix and the Pauli X matrix, respectively, and ${O}_{{2}^{n}}$ denotes the ${2}^{n}\times {2}^{n}$ zero matrix. The proof of the recurrence relation is given in Supplementary Note 1. The indices $\left\{{\upxi }_{k}\right\}$ in Eq. (9) can be determined once ${U}_{{2}^{n}}^{\mathrm{ent}}$ is obtained. Finally, the inner product $\langle {\psi }^{i}|{\psi }^{j}\rangle$ can be calculated by ${\sum }_{k}{f}_{k}^{*}\left({{\varvec{s}}}^{(i)}\right){f}_{k}\left({{\varvec{s}}}^{(j)}\right)$.

### Details of computational resources

Our quantum AI simulator based on a hybrid CPU–FPGA system is implemented on the Amazon Web Services (AWS) Elastic Computing Cloud (EC2) platform, in which AWS EC2 F1 instances of AMD Xilinx FPGA hardware are accessible. In particular, we used the f1.2xlarge instance size, which has 1 FPGA, 8 vCPUs, and 122 GB of memory. More specifically, we used AMD Xilinx Virtex™ UltraScale + ™ VU19P FPGA and Intel Xeon™ E5-2686 v4 with a base clock speed of 2.3 GHz as vCPU. The details of our FPGA architecture and block diagrams are provided in Supplementary Note 2.

### Machine learning

Here we provide the details of our ML models. Preprocessing was applied to the original data to make them suitable for quantum angle encoding: PCA was used to reduce the dimension of the $28\times 28$ original image data to $d$-dimensional input vectors ${{\varvec{x}}}^{(i)}\in {\mathbb{R}}^{d}$ (where $d$ was varied from 4 to 780), which were then transformed such that ${{\varvec{x}}}^{(i)}\in [-\mathrm{1,1}]$. In the training of support vector classifiers, hinge loss was used for the loss function. Throughout the paper, the regularization parameter $C$ for soft margin SVM^47^ was set to 1.0 for both classical and quantum ML models. For the multiclass classification task shown in Fig. 4, a one-versus-rest strategy was employed.

To compare the performance of our quantum kernel with the classical counterpart, we used the Gaussian kernel, which is given by $\mathrm{exp}\left(-\gamma {\Vert {{\varvec{x}}}^{(i)}-{{\varvec{x}}}^{(j)}\Vert }^{2}\right)$, with $\gamma$ being a hyperparameter. To obtain the optimal test accuracy, we performed a grid search over the bandwidth.

11 $$\gamma \in \left\{0.001, 0.1, 0.25, 0.5, 0.75, 1, 1.25, 2.5, 3.75, 5, 6.25, 7.5, 8.75, 10, 50, 100, 1000\right\}.$$

It is also possible to introduce a hyperparameter in our quantum feature map $\left|{\Psi }^{\mathrm{BPS}}\left({\varvec{x}}\right)\rangle \right.$. In this work, we consider that the input vector ${\varvec{x}}$ can be scaled by $\uplambda$ (i.e., ${{\varvec{x}}}^{(i)}\leftarrow\uplambda {{\varvec{x}}}^{(i)}$), which is similar to an approach by recent work^15^. Thus, we performed a grid search over the scaling parameter.

12 $$\uplambda \in \left\{0.001, 0.1, 0.25, 0.5, 0.75, 1, 1.25, 2.5, 3.75, 5, 6.25, 7.5, 8.75, 10, 50, 100, 1000\right\}.$$

The effect of the scaling parameter $\uplambda$ was somewhat different from that of $\gamma$. In particular, we found that, for binary classification, the case of $\uplambda =1$ typically gave the near-optimal performance (see also Supplementary Note 3), implying that our quantum kernel gave a reasonable performance without introducing any hyperparameter. Nonetheless, to further optimize the value for $\uplambda$, we narrowed the range for $\uplambda$ and performed another grid search over the scaling parameter

$$\uplambda \in \{0.5, 0.55, 0.6, 0.65, 0.7, 0.75, 0.8, 0.85, 0.9, 0.95, 1, 1.05,$$

13 $$1.1, 1.15, 1.2, 1.25, 1.3, 1.35, 1.4, 1.45, 1.5, 1.55, 1.6\}.$$

We found that the test accuracy was slightly improved from 0.870 to 0.875 in binary classification (see also Supplementary Note 4) and that the use of the scaling parameter $\uplambda$ played an important role in multiclass classification.

## Supplementary Information

Supplementary Information.

## Data Availability

All the datasets used in the present study are publicly available at https://github.com/zalandoresearch/fashion-mnist. We cited the reference of the data in the manuscript.
