# Supplementary material for: Quantum AI simulator using a hybrid CPU–FPGA approach
Source: Sci Rep. 2023 May 12;13:7735. doi: 10.1038/s41598-023-34600-2 (PMC10182082; doi:10.1038/s41598-023-34600-2)
Supplement: Supplementary file 1 — Supplementary Information. [file 41598_2023_34600_MOESM1_ESM.pdf]

Supplementary Information for

"Quantum AI simulator using a hybrid CPU–FPGA approach"

Teppei Suzuki[1,*], Tsubasa Miyazaki[1], Toshiki Inaritai[1], and Takahiro Otsuka[1]

[1] *Research and Development Center, SCSK Corporation, Toyosu Front, 3-2-20 Toyosu, Koto-ku, Tokyo 135-8110, Japan*

Supplementary Note 1. Recurrence relation for an $\boldsymbol{n}$-qubit entanglement operation matrix

We discuss how we can calculate a unitary matrix $U_{2^n} = \prod_{q=1}^{n-1} \mathbf{CNOT}_{q,q+1}$, without actually conducting tensor product operations. The matrix U_{2^n} is a sparse matrix that can be recursively obtained using Proposition 1.

Proposition 1 (Recurrence relation). *Let* $n \in \mathbb{N}$. *Let* $\{U_{2^n}\}$ *and* $\{Y_{2^n}\}$ *be sequences of square matrices such that*

$$U_{2^{n+1}} := (I_{2^{n-1}} \otimes \mathbf{CNOT})(U_{2^n} \otimes \mathbf{ID}),$$
$$Y_{2^{n+1}} := (I_{2^{n-1}} \otimes \mathbf{CNOT})(Y_{2^n} \otimes \mathbf{ID}),$$

(A1)

where $\mathbf{CNOT}$ *and* $\mathbf{ID}$ *are the matrices representing the controlled* NOT *gate and the identity gate, respectively and* I_{2^n} *denotes the* $2^n \times 2^n$ *identity matrix, with* $I_{2^0} := 1$*; and let* U_2 *and* Y_2 *be defined by the* 2×2 *identity matrix and the Pauli X matrix, respectively:*

$$U_2 := I_2 = \begin{bmatrix} 1 & 0 \\ 0 & 1 \end{bmatrix}; \quad Y_2 := X = \begin{bmatrix} 0 & 1 \\ 1 & 0 \end{bmatrix}.$$

(A2)

Then $U_{2^{n+1}}$ *and* $Y_{2^{n+1}}$ *can be calculated by recursion*

$$U_{2^{n+1}} = \begin{bmatrix} U_{2^n} & O_{2^n} \\ O_{2^n} & Y_{2^n} \end{bmatrix} \quad (n \geq 1),$$

$$Y_{2^{n+1}} = \begin{bmatrix} O_{2^n} & U_{2^n} \\ Y_{2^n} & O_{2^n} \end{bmatrix} \quad (n \geq 1),$$

(A3)

where O_{2^n} *denotes the* $2^n \times 2^n$ *zero matrix.*

Proof. We prove the statement by induction on n.

Step I. Base case ($n = 1$):

$$U_4 = (1 \otimes \mathbf{CNOT})(U_2 \otimes \mathbf{ID}) = (1 \otimes \mathbf{CNOT})(I_2 \otimes \mathbf{ID}) = \begin{bmatrix} I_2 & O \\ O & X \end{bmatrix} \begin{bmatrix} I_2 & O \\ O & I_2 \end{bmatrix} = \begin{bmatrix} U_2 & O_2 \\ O_2 & Y_2 \end{bmatrix}, \tag{A4}$$

and

$$Y_4 = (1 \otimes \mathbf{CNOT})(Y_2 \otimes \mathbf{ID}) = (1 \otimes \mathbf{CNOT})(X \otimes \mathbf{ID}) = \begin{bmatrix} I_2 & O \\ O & X \end{bmatrix} \begin{bmatrix} O & I_2 \\ I_2 & O \end{bmatrix} = \begin{bmatrix} O & I_2 \\ X & O \end{bmatrix} = \begin{bmatrix} O_2 & U_2 \\ Y_2 & O_2 \end{bmatrix}. \tag{A5}$$

Hence, the statement is true for $n = 1$. Note that U_4 is the **CNOT** gate itself.

Step II. Induction step: we assume that the statement holds for some natural number k. For $n = k+1$, we have

$$\begin{aligned} U_{2^{k+2}} &= \left(I_{2^k} \otimes \mathbf{CNOT}\right)\left(U_{2^{k+1}} \otimes \mathbf{ID}\right) \\ &= \begin{bmatrix} I_{2^{k-1}} \otimes \mathbf{CNOT} & O_{2^{k+1}} \\ O_{2^{k+1}} & I_{2^{k-1}} \otimes \mathbf{CNOT} \end{bmatrix} \begin{bmatrix} U_{2^k} \otimes \mathbf{ID} & O_{2^{k+1}} \\ O_{2^{k+1}} & Y_{2^k} \otimes \mathbf{ID} \end{bmatrix} \\ &= \begin{bmatrix} \left(I_{2^{k-1}} \otimes \mathbf{CNOT}\right)\left(U_{2^k} \otimes \mathbf{ID}\right) & O_{2^{k+1}} \\ O_{2^{k+1}} & \left(I_{2^{k-1}} \otimes \mathbf{CNOT}\right)\left(Y_{2^k} \otimes \mathbf{ID}\right) \end{bmatrix} = \begin{bmatrix} U_{2^{k+1}} & O_{2^{k+1}} \\ O_{2^{k+1}} & Y_{2^{n+1}} \end{bmatrix}, \end{aligned} \tag{A6}$$

and

$$\begin{aligned} Y_{2^{k+2}} &= \left(I_{2^k} \otimes \mathbf{CNOT}\right)\left(Y_{2^{k+1}} \otimes \mathbf{ID}\right) \\ &= \begin{bmatrix} I_{2^{k-1}} \otimes \mathbf{CNOT} & O_{2^{k+1}} \\ O_{2^{k+1}} & I_{2^{k-1}} \otimes \mathbf{CNOT} \end{bmatrix} \begin{bmatrix} O_{2^{k+1}} & U_{2^k} \otimes \mathbf{ID} \\ Y_{2^k} \otimes \mathbf{ID} & O_{2^{k+1}} \end{bmatrix} \\ &= \begin{bmatrix} O_{2^{k+1}} & \left(I_{2^{k-1}} \otimes \mathbf{CNOT}\right)\left(U_{2^k} \otimes \mathbf{ID}\right) \\ \left(I_{2^{k-1}} \otimes \mathbf{CNOT}\right)\left(Y_{2^k} \otimes \mathbf{ID}\right) & O_{2^{k+1}} \end{bmatrix} = \begin{bmatrix} O_{2^{k+1}} & U_{2^{k+1}} \\ Y_{2^{k+1}} & O_{2^{k+1}} \end{bmatrix}. \end{aligned} \tag{A7}$$

We can see that the statement holds true for $n = k+1$. Hence, the statement holds for all natural numbers $n \geq 1$. □

Note that matrices U_{2^n} and Y_{2^n} are sparse; and the sparsity pattern visualization for U_{2^n} and Y_{2^n} is shown Supplementary Figure 1. In the calculation of our quantum feature map, we only need the index for non-zero entry in each row vector of U_{2^n}.

a

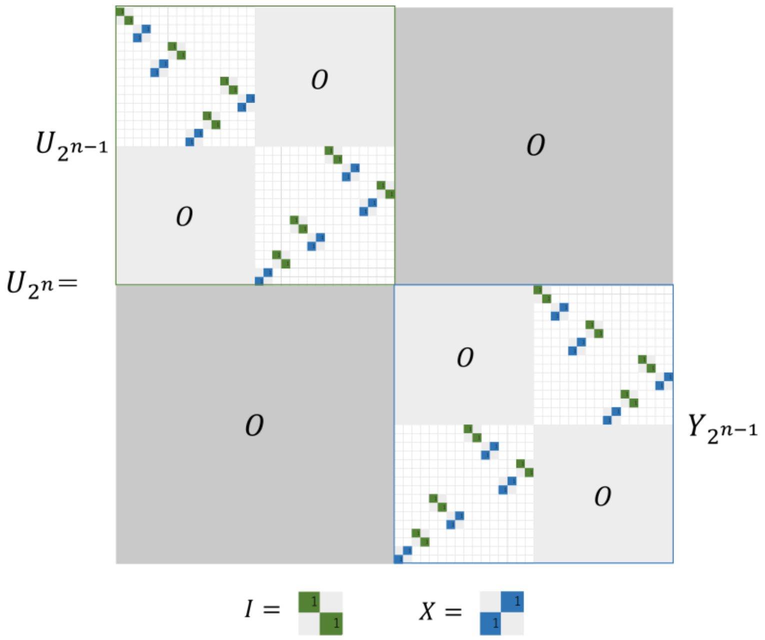

b

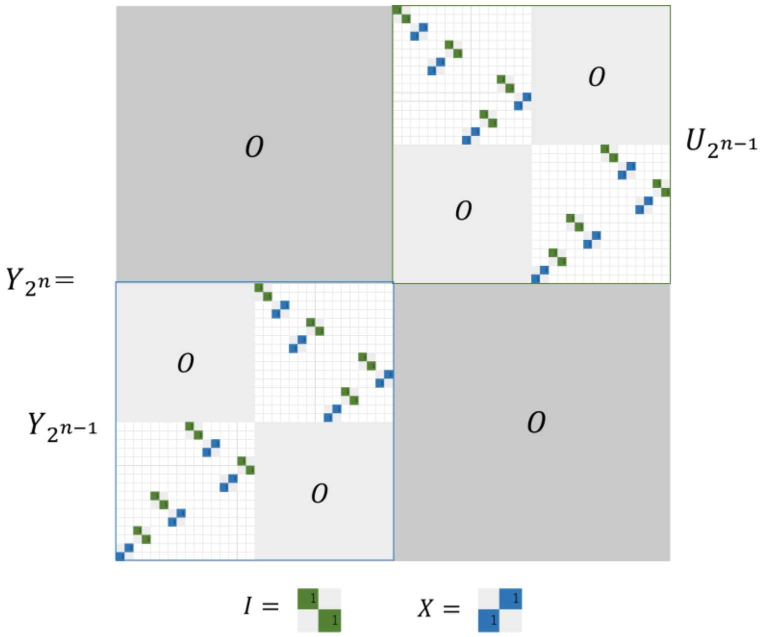

Supplementary Figure 1: Sparsity pattern visualization for U_{2^n} and Y_{2^n}. Shown are matrices U_{2^n} (a) and Y_{2^n} (b) in the case of $n = 6$ (i.e., U_{64} and Y_{64}). Values of one that are originally belonging to matrices I and X are represented by green and blue, respectively, while other entries are zero. The matrix U_{2^n} represents an n-qubit entanglement operation $\prod_{q=1}^{n-1} \mathbf{CNOT}_{q,q+1}$. The matrix U_{2^n} has a property $\mathrm{Tr}(U_{2^n}) = 2$ for all n.

Supplementary Note 2. FPGA architecture and block diagrams for computing the quantum kernel

In this section, we describe the details of our FPGA architecture and block diagrams for computing the quantum kernel. The overview of the quantum kernel implementation is shown in Supplementary Figure 2. Our quantum AI simulator based on a hybrid CPU–FPGA approach is implemented on Amazon Web Services cloud platform, in which Amazon EC2 F1 instances of Xilinx FPGA hardware are accessible. First, PCA-reduced features are sent from CPU (the host) to FPGA via PCIe. Second, the sine and cosine of the input angles are computed using the CORDIC algorithm [S1] (Supplementary Figure 3). Third, the unitary matrices U and V (which are defined by Eq. (7) in the text) are computed (Supplementary Figures 4 and 5). Fourth, the quantum feature map is calculated using the unitary matrices U and V, as well as an efficient implementation of n-qubit quantum entanglement (Supplementary Figure 6). Fifth, the square of the norm of the inner product is obtained (Supplementary Figure 7). Finally, the data is sent back to the host (CPU). In Supplementary Table 1, we give the details hardware utilizations for the quantum kernel implementation.

- Supplementary Figure 2: Scheme for the quantum kernel implementation.
- Supplementary Figure 3: Schematic block diagram for the module that computes sine and cosine functions of the input angles.
- Supplementary Figure 4: Block diagram for the module that computes the unitary matrices U_q and V_q using the sine and the cosine values.
- Supplementary Figure 5: Schematic block diagram for the module that computes the tensor product.
- Supplementary Figure 6: Schematic block diagram for the module that computes the quantum feature map.
- Supplementary Figure 7: Block diagram for the module that computes the square of the norm of the inner product.
- Supplementary Table 1: Hardware utilizations for the quantum kernel implementation.

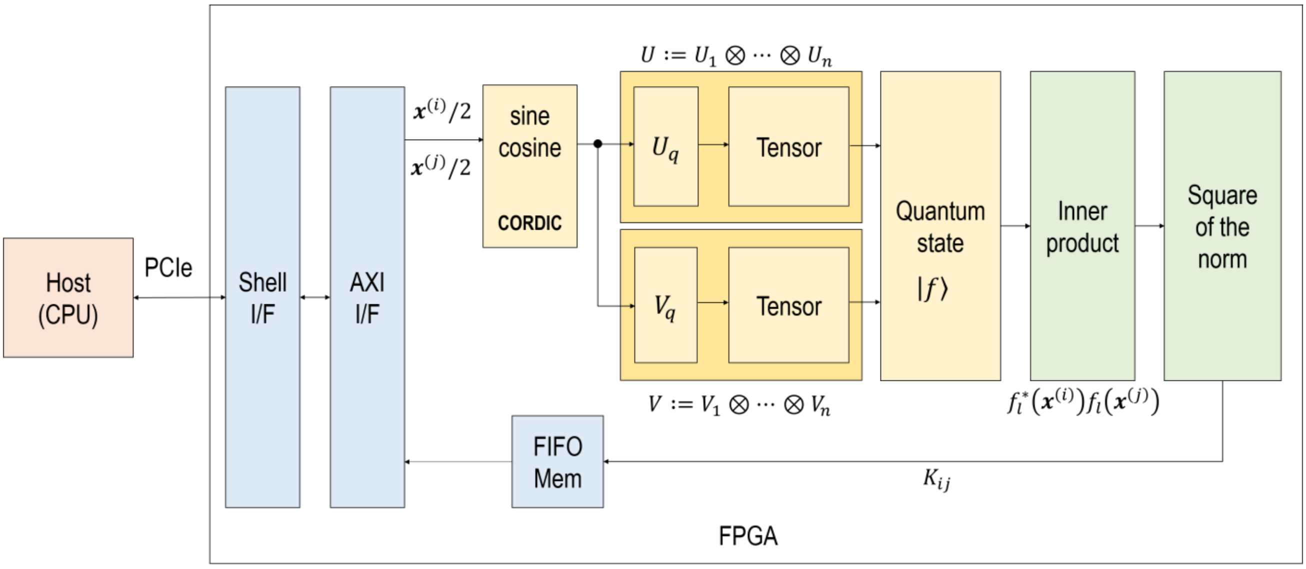

Supplementary Figure 2: Scheme for the quantum kernel implementation. Our quantum AI simulator based on a hybrid CPU–FPGA system is implemented on Amazon Web Services cloud platform, in which Amazon EC2 F1 instances of Xilinx FPGA hardware are accessible. PCA-reduced features are sent from CPU (the host application) to FPGA via PCIe. The quantum feature map can be obtained in the following steps (denoted by the yellow boxes): First, the sine and cosine of the input angles are computed using the CORDIC algorithm. Second, the unitary matrices U and V (which are defined by Eq. (7) in the text) are computed. Third, the quantum feature map is calculated using the unitary matrices U and V, as well as an efficient implementation of n-qubit quantum entanglement. The square of the norm of the inner product is computed (denoted by the green boxes), generating the quantum kernel entry K_{ij}. Finally, the data is sent back to the host.

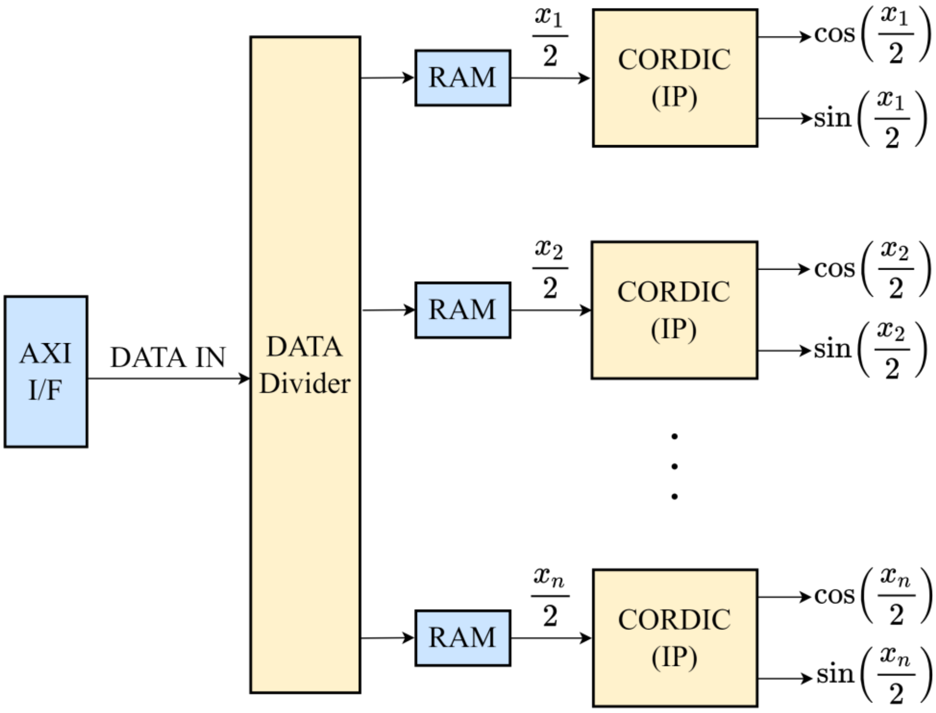

Supplementary Figure 3: Schematic block diagram for the module that computes sine and cosine functions of the input angles. Data input (PCA-reduced features) in CPU are sent and recognized as a streaming data by AXI, which is an interface between the host application (CPU) and FPGA. The data are divided into units consisting of $\frac{x_1}{2}, \frac{x_2}{2}, \cdots, \frac{x_n}{2}$ (via the *data divider*); and each value is stored in RAM. Then the stored data are read out and the sine and cosine of the input angles are computed using the CORDIC algorithm.

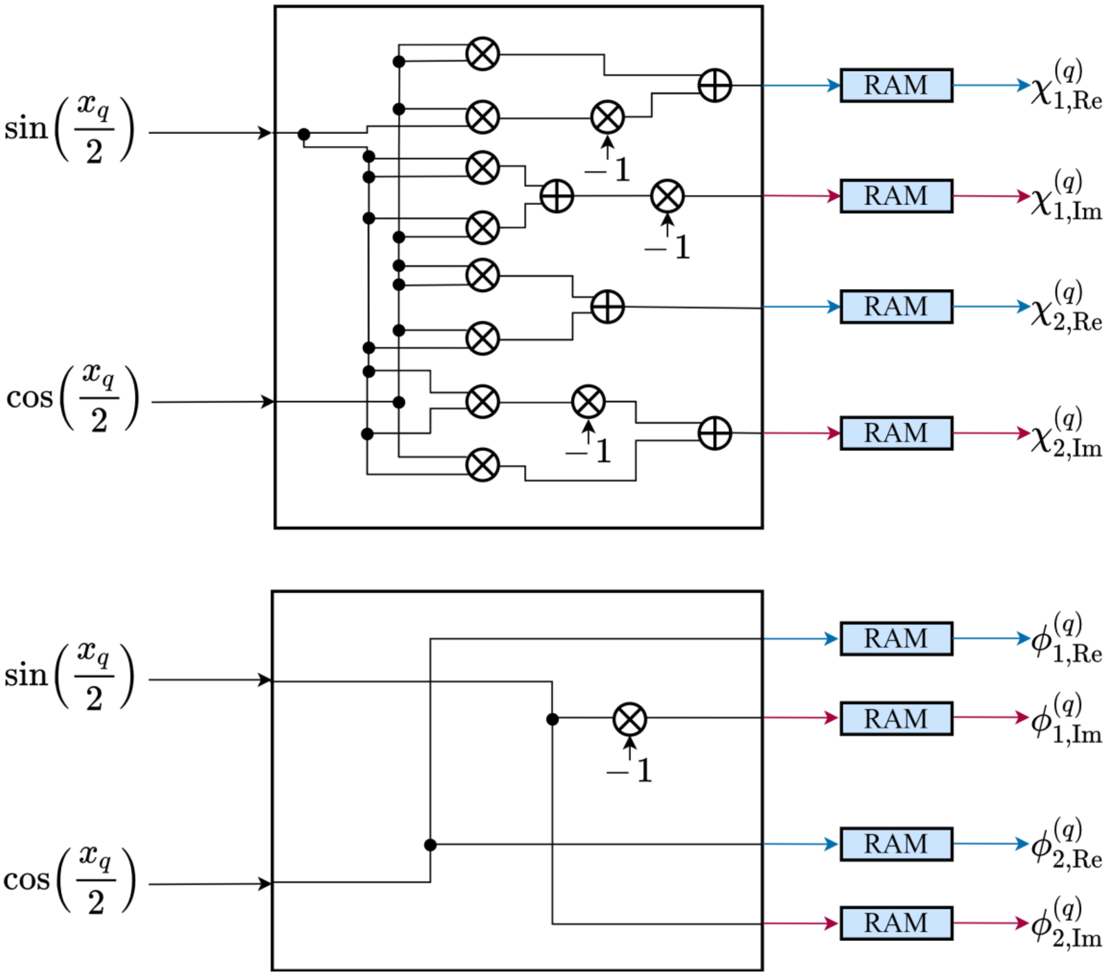

Supplementary Figure 4: Block diagram for the module that computes the unitary matrices U_q and V_q using the sine and the cosine values. (Top) Complex-valued entries $\chi_1^{(q)}$ and $\chi_2^{(q)}$ are generated using the sine and cosine of the input data in order to obtain the first column vector of each 2×2 unitary matrix $U_q = R_y(x_q) R_z(x_q) H$; the prefactor associated to the Hadamard gate will be multiplied later in the calculation. (Bottom) Complex-valued entries $\phi_1^{(q)}$ and $\phi_2^{(q)}$ are generated in order to obtain the diagonal elements of each 2×2 unitary matrix $V_q = R_z(x_q)$. Re and Im in the figure denote the real and imaginary parts of a complex number, respectively.

Supplementary Figure 5 (*continued on next page*). (*Caption shown on next page.*)

a

b

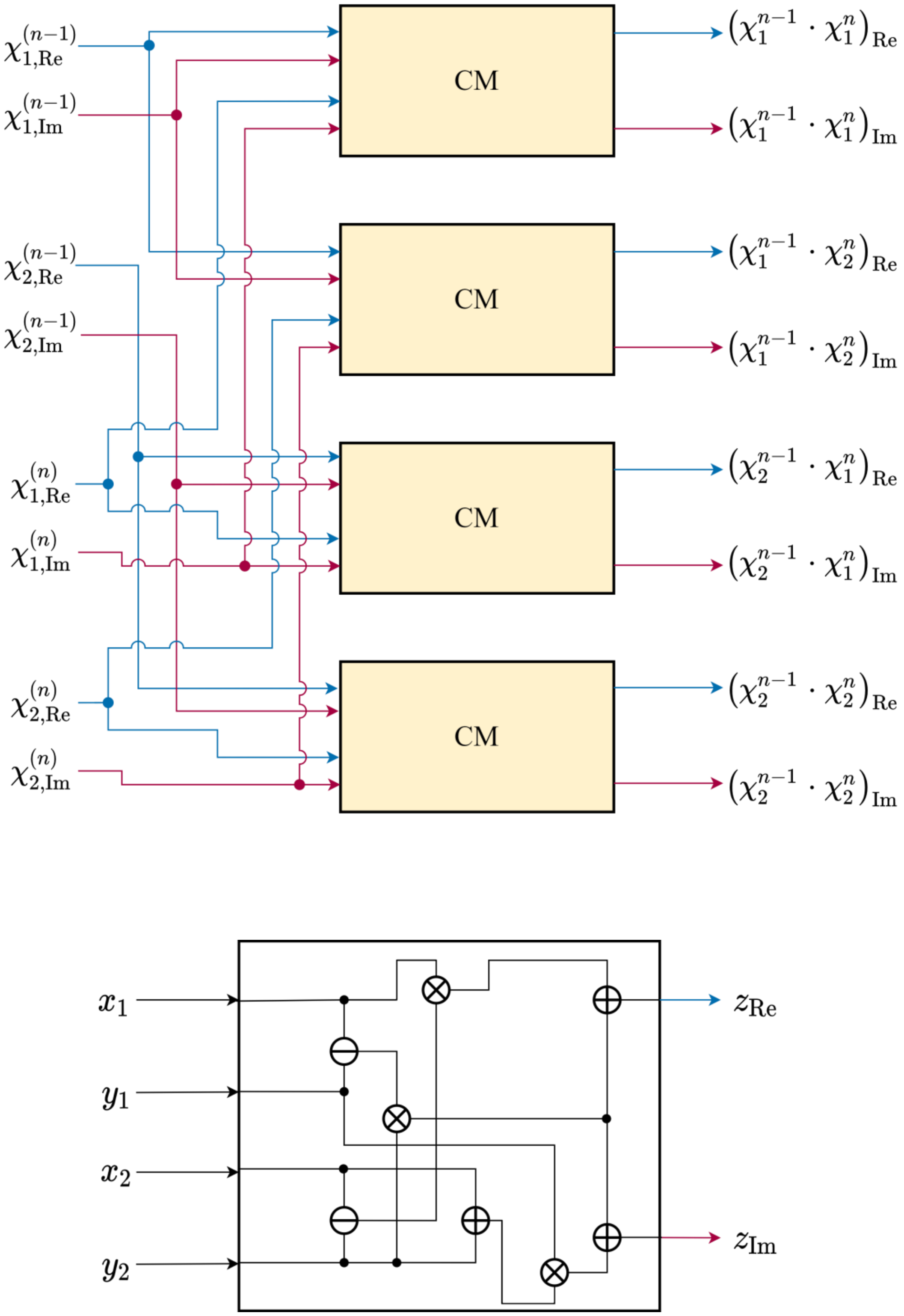

Supplementary Figure 5 (*continued*).

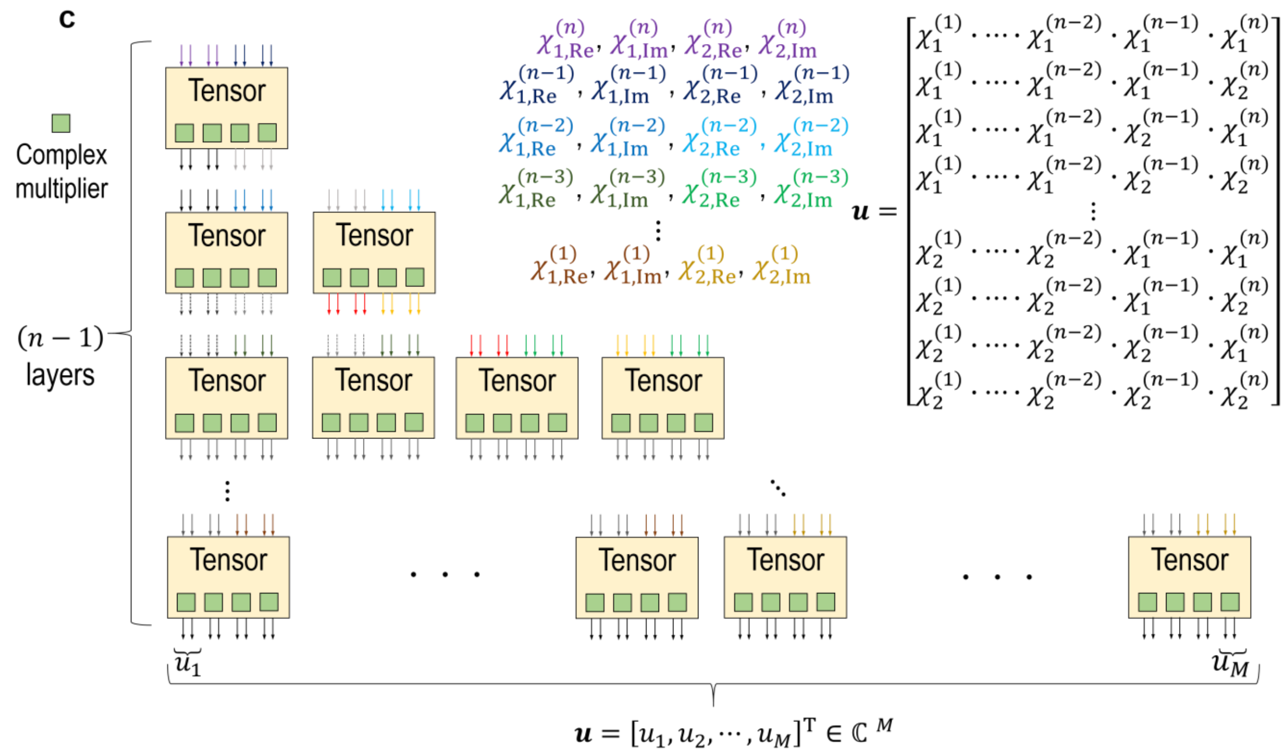

Supplementary Figure 5: Schematic block diagram for the module that computes the tensor product. Re and Im denote the real and imaginary parts of a complex number, respectively. (a) Schematic block diagram for a tensor-product subunit. Shown is an example for the input $(\chi_1^{(n-1)}, \chi_2^{(n-1)})$ and $(\chi_1^{(n)}, \chi_2^{(n)})$, which is the first layer in panel c. CM denotes complex multiplier, which is given in panel b. (b) Block diagram for complex multiplier: $z = (x_1 + iy_1) \cdot (x_2 + iy_2)$. (c) Schematic representation of the tensor product operation in FPGA. The tensor-product subunits are denoted by the yellow boxes, each of which contains four complex multipliers (see also part a). In our quantum feature map, only the first column of the matrix $U = U_1 \otimes \cdots \otimes U_n$ is needed; thus, it can be computed by Eq. (8), which is also shown in the right corner of panel c. From the schematic diagram depicted by panel c, we can see that the number of complex multipliers for this module is $4 \cdot (2^{n-1} - 1)$. During the calculation, a prefactor of $1/2$, which is associated to the Hadamard gate, is multiplied every two steps (if the total number of the steps is odd, then a prefactor of $1/2 = (1/\sqrt{2}) \cdot (1/\sqrt{2})$ is multiplied at the very end of the calculation of the square of the norm of the inner product (see also Supplementary Figure 7)). In a similar manner, we can also obtain $V = V_1 \otimes \cdots \otimes V_n$, where V_q is given by the rotation operator gate $R_z(x_q)$; in this case, only the operations involving the diagonal elements of V are needed and there is no need for the prefactor adjustment.

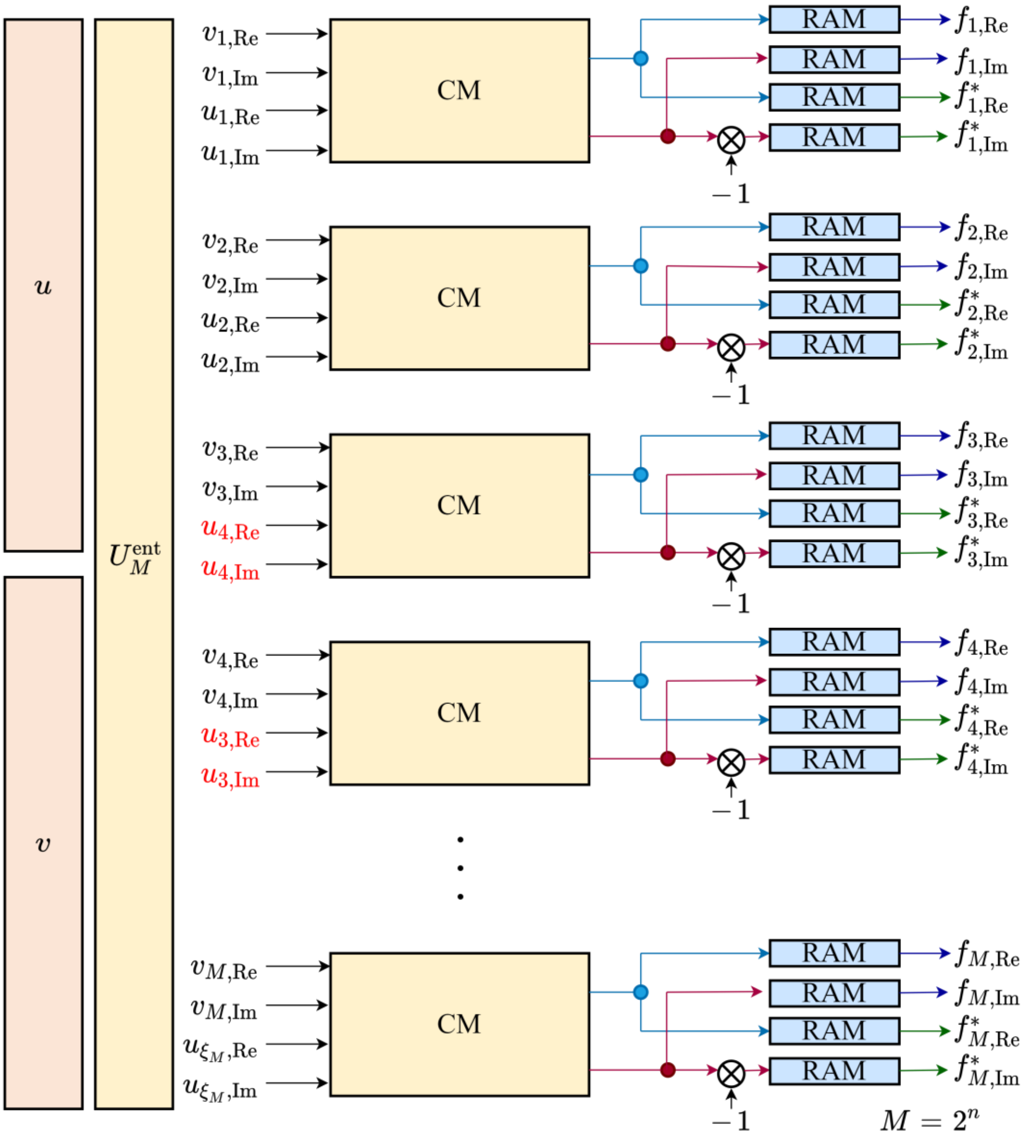

Supplementary Figure 6: Schematic block diagram for the module that computes the quantum feature map. On the basis of Eq. (9), the quantum state $\boldsymbol{f} \in \mathbb{C}^{2^n}$ can be computed using the unitary matrices U and V (which are obtained by the modules described in Supplementary Figures 4 and 5). The vector $\boldsymbol{u}$ is the first column of the unitary matrix U; and the vector $\boldsymbol{v}$ is the diagonal element of the diagonal matrix V. In our quantum feature map defined by Eq. (7), the role of n-qubit entanglement $\prod_{q=1}^{n-1} \mathbf{CNOT}_{q,q+1}$ can be viewed as 'rearranging' the elements of the vector $\boldsymbol{u}$ in accordance with the matrix U_{2^n} defined in Supplementary Figure 1a. For instance, $f_3 = v_3 u_{\xi_3} = v_3 u_4$ and $f_4 = v_4 u_{\xi_4} = v_4 u_3$, which are indicated by red in the figure. Such technique leads to an efficient implementation of the quantum state including quantum entanglement. The complex conjugate of the state vector $\boldsymbol{f}$ can also be computed by changing the sign of the imaginary part of $\boldsymbol{f}$. The quantum states can thus be obtained for a pair of ith and jth samples, which are denoted by the green and the blue arrows, respectively. Re and Im in the figure denote the real and imaginary parts of a complex number, respectively. For the block diagram of complex multiplier (CM), see panel b of Supplementary Figure 5.

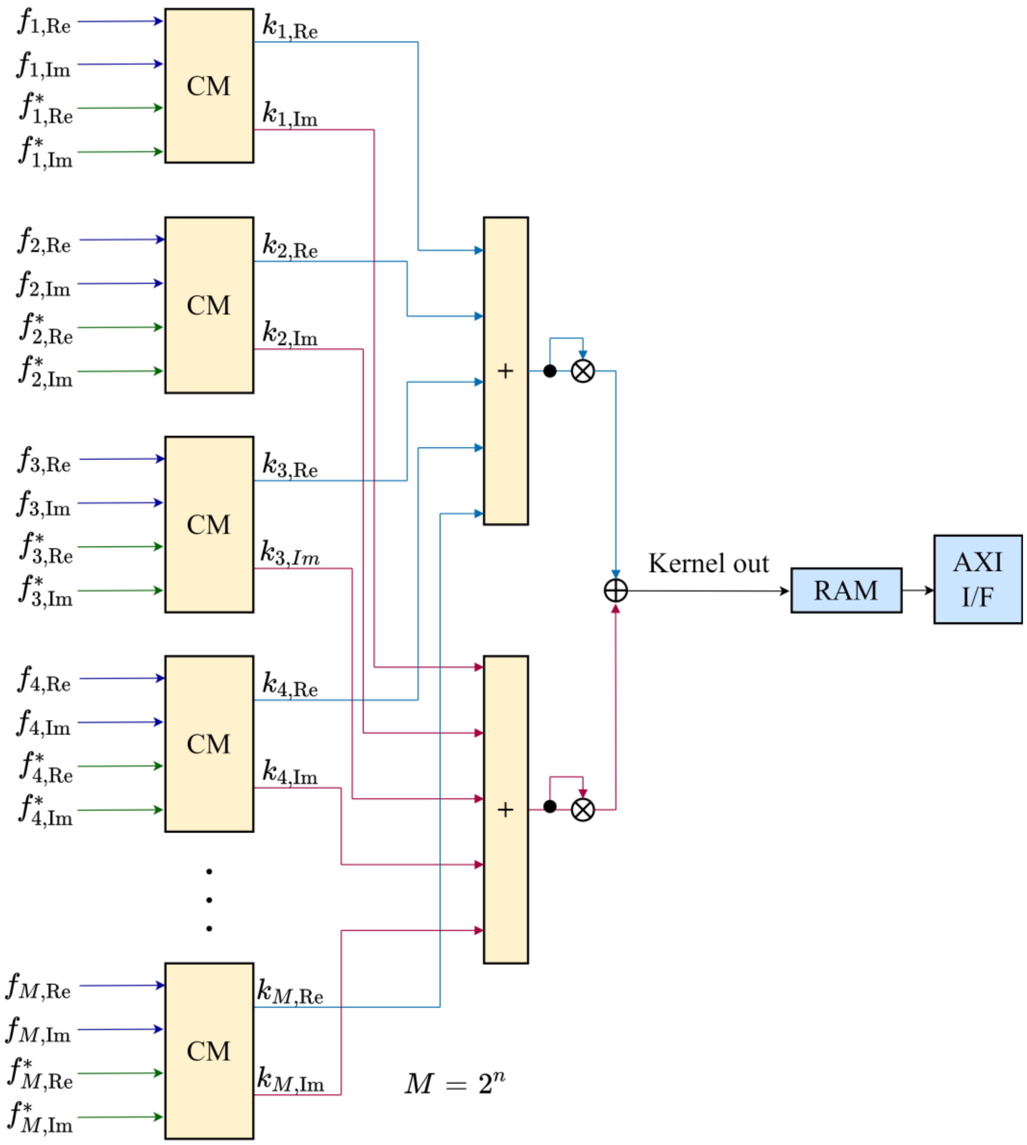

Supplementary Figure 7: Block diagram for the module that computes the square of the norm of the inner product. The inner product can be obtained using complex multipliers: $k_l := f_l^*(\boldsymbol{x}^{(i)}) f_l(\boldsymbol{x}^{(j)}) \in \mathbb{C}$ $(l = 1, \cdots, M)$ for a pair of ith and jth samples, which are denoted by the green and the blue arrows, respectively. Then the quantum kernel entry K_{ij} is given by the sum of $(\sum_l \mathrm{Re}[k_l])^2$ and $(\sum_l \mathrm{Im}[k_l])^2$, which are indicated by the light-blue and the red arrows, respectively. The data are stored in RAM and sent back to the host. For the block diagram of complex multiplier (CM), see part b of Supplementary Figure 5.

Supplementary Table 1: Hardware utilizations for the quantum kernel implementation

Chip	XCVU9P (AWS F1 instance)	XCVU9P (AWS F1 instance)
Number of qubits	2	6
Train size	1024	1024
LUT	175077/1180984 (15%)	237681/1180984 (20%)
LUTRAM	17335/591440 (3%)	57360/591440 (10%)
FF	250576/2364480 (11%)	327521/2364480 (14%)
BRAM	365.5/2160 (17%)	515/2160 (24%)
URAM	43/960 (4%)	43/960 (4%)
DSP	61/6840 (1%)	1154/6840 (17%)
Clock frequency (MHz)	250	250

Abbreviations: LUT, look-up table; RAM, random access memory; FF, flip flop; BRAM, block RAM; URAM, ultraRAM; DSP, digital signal processor.

Supplementary Note 3. Grid search over the hyperparameters for the classical and quantum kernels

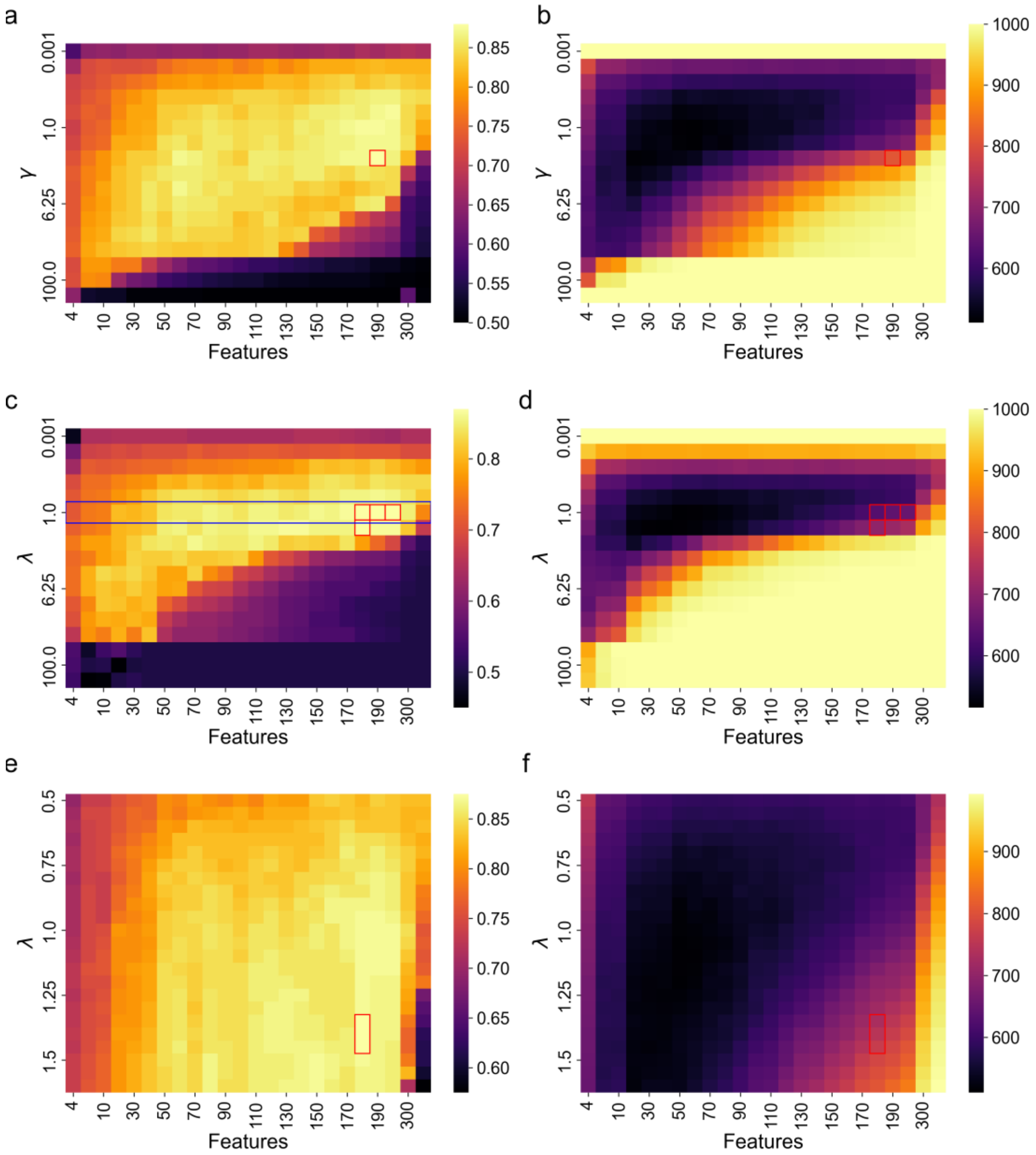

Supplementary Figure 8: Grid search over the hyperparameters for the classical and the quantum SVM on binary classification as a function of features. Fashion-MNIST dataset was used. The hyperparameters giving the optimal test accuracy are indicated by the open red square. (a) Grid search over the hyperparameter γ for the Gaussian kernel: (a) test accuracy and (b) the number of the corresponding support vectors. Grid search over the scaling parameter λ for the quantum kernel. (c) test accuracy and (d) the number of the corresponding support vectors. Note that, in our quantum kernel (panel c), the case of $\lambda = 1$ typically gave the near-optimal performance (indicated by the open blue rectangle), implying that our quantum kernel gave a reasonable performance without introducing any hyperparameter. Nonetheless, to further optimize the value for λ, we narrowed the range for λ and performed another grid search over the scaling parameter: (e) test accuracy and (f) the number of the corresponding support vectors. A slightly better was obtained.

Supplementary Note 4. Details of numerical simulations

We used the scikit-learn library [S2] for support vector machines. In order to compare our FPGA implantation with a quantum computing simulator, we used Qiskit, an open source software development kit [S3] (ver. 0.31.0) and Qiskit Aer (ver. 0.9.1) to perform quantum computing simulations in order to generate the quantum kernel in Fig. 2d in the main text.

Supplementary References

[S1] Volder, J. E. The CORDIC trigonometric computing technique. *IRE Trans. Electron. Comput.* **3**, 330–334 (1959).

[S2] Pedregosa, F. *et al.* Scikit-learn: Machine learning in Python. *J. Mach. Learn. Res.* **12**, 2825–2830 (2011).

[S3] Aleksandrowicz, G. *et al.* Qiskit: An open-source framework for quantum computing. https://github.com/qiskit (Accessed September 13, 2022).
